# Association between physical exercise and all-cause and CVD mortality in patients with diabetes: an updated systematic review and meta-analysis

**DOI:** 10.4314/ahs.v22i3.27

**Published:** 2022-09

**Authors:** Xinmin Liu, Zhen Wu, Ning Li

**Affiliations:** Sports and Health University of Heze University, Heze, China

**Keywords:** physical activity, mortality, CVD, diabetes

## Abstract

**Objectives:**

Physical activity is recommended in guidelines for treatment for diabetes, but the association between physical activity and mortality among diabetic patients has not been extensively studied.

**Methods:**

Databases were searched from inception to July 10, 2020. Prospective studies were selected to evaluate the association between physical activity and risk for total and cardiovascular diseases (CVD) mortality among diabetic patients. Data were pooled using random-effect model to calculate the relative risks (RRs) with 95% confidence intervals (CIs).

**Results:**

We included 16 eligible studies involving with 155,203 diabetic participants and 13,821 cases of death. Our study suggested that physical activity in diabetic patients may decrease risk for all-cause (RR 0.57, 95% CI 0.49–0.67) and CVD mortality (RR 0.55, 95% CI 0.34–0.68). The summary RR for CVD events was 0.65 (95% CI 0.41–1.03). Furthermore, the reductions in all-cause mortality were more significant in diabetic patients with old age (> 60 years) (RR 0.46, 95% CI 0.29 -0.75), higher body mass index (BMI ≥ 28) (RR 0.53, 95% CI 0.42–0.69) and shorter duration of diabetes (RR 0.45, 95% CI 0.24–0.84).

**Conclusion:**

Physical activity reduced the risk of total and CVD mortality among patients with diabetes, in particular in diabetic patients with old age (> 60 years), obesity and shorter duration of diabetes.

## Background

Diabetes was recognized as a substantial threat to public health with the fastest increasing morbidity worldwide 1. It was reported that 415 million people were estimated to have diabetes in 2015, and will increase to 642 million by 2040 2,3. Patients with diabetes are at high risk for adverse outcomes from its macro vascular and micro vascular complications, which account for more than 2 million deaths every year 4 and constitute the seventh most common cause of disability worldwide 5. The absolute number of deaths from diabetes increased between 2006 and 2016 by 31.1% 6. Among adults in China, diabetes was associated with 2-fold increased mortality compared with adults without diabetes 7. Cardiovascular diseases (CVD) is the most common cause of morbidity and mortality among subjects with type 2 diabetes mellitus (T2DM) 8. Body mass index (BMI) is an independent risk factor of diabetes and CVD 9, which were closely related with physical exercise. Physical activity is important in the prevention of the development of T2DM in people with impaired glucose tolerance (IGT) and for the control of glycaemia and related CVD complications 10,11. Several studies have indicated that high leisure-time physical activity is associated with reduced total and CVD mortality among patients with diabetes 12–17. However, results were not consistent 14,17. In diabetic patients with heart diseases or other serious complication, physical activity may worsen their health conditions. Furthermore, how diabetes severity, patients' age or BMI influence this association has not been extensively studied. Several newly studies on the association between physical activity and mortality have been published. The aim of this study was to examine associations of physical activity with risk total and CVD mortality among diabetic patients. Furthermore, we analyzed the association in diabetic patients with different characteristics such as age, BMI, glycated haemoglobin A1C (HbA1c), types and duration of diabetes.

## Materials and methods

This meta-analysis is reported according to Meta-analysis Of Observational Studies in Epidemiology (MOOSE) Reporting Guidelines 18.

### Search strategy

A systematic literature search for relevant studies was conducted in the databases of Pub Med and EMBASE from inception to January 15th, 2020. The search strategy was as follows: (((((((Diabetes Mellitus [MeSH Terms])) OR (diabetes)) OR (type 2 diabetes)) OR (type 1 diabetes) [All Fields])) AND ((((physical activity[All Fields])) OR (exercise)) OR (sports))) AND ((((death[All Fields])) OR (mortality)) OR (fetal)). In addition, we reviewed the references from relevant articles to identify additional relevant studies. Authors were contacted and requested to provide further data if required.

### Study selection

The following inclusion criteria were required to be eligible for the meta-analysis: (1) cohort, prospective or longitudinal study with more than 5-year follow up; (2) diabetic patients with active and inactive physical activity; (3) reported relative risk (RR) estimates of mortality, such as relative risks (RR), odds ratios (OR), hazard ratios (HR) or incidence with 95% confidence intervals (CIs) for 2 or more categories of physical activity. In multiple same-population studies, we selected and included the one study with longest follow-up time.

### Data extraction

For each eligible study, we recorded the following data: name of the first author, year of publication, study name, study location, participants, age at baseline, and measurement for physical activity, number of diabetes and death, years of follow-up and outcomes.

### Statistical Methods

The pooled analyses were performed using the random-effects model to calculate RRs with 95% CIs). If several estimates were reported in the same article, we chose the most fully adjusted RR of the top category vs. the lowest category of physical activity. If the reference category used in the analyses was not the lowest category, we used the method described was by Hamling et al 19 to convert risk estimates. Study quality was assessed using Newcastle-Ottawa scale (NOS) 20. Heterogeneity was quantified using the I2 test, where I2 > 50% indicated significant heterogeneity 21. Publication bias was evaluated by the Egger's and Begg's test 22,23. Sensitivity analyses were performed to assess the robustness of the findings by omitting each study from the analyses and then summarized the remains. Subgroup analyses were conducted to investigate the impact of age, study location, number of participants and case, follow-up of cohort studies, HbA1c, type and duration of diabetes on the association between physical activity and risk of total and CVD mortality among diabetes.

All the analyses were conducted using Stata statistical software (version 16.0). A 2-sided P value of less than 0.05 was considered statistically significant.

## Results

### Study Selection

**Figure S1:**
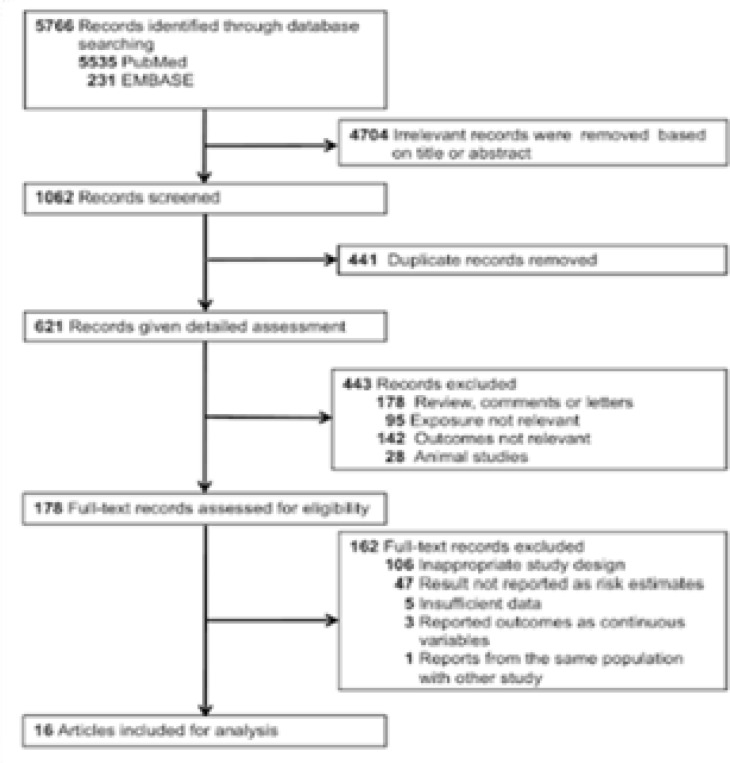
Study selection.

Of 5,766 studies identified by the initial search, 178 were selected for full-text review; 162 of these were excluded, leaving 16 (Figure S1). Two studies were from the same study 16,24, and we included the one with larger sample size 16.

### Study characteristics

After ineligible studies were excluded, 16 cohort studies were included in our meta-analysis. (Descriptive characteristics of studies and outcomes are shown in Table S1)^12^,^13^,^29^–^34^,^14^–^17^,^25^–^28^. It involved with 155,203 diabetic participants and 13,821 cases of death. Participants were aged 25 to 80 years, with more than half being middle-aged or older. The duration of cohort studies ranged from 5.7 to 23.8 years, with a median year of 8.7. Results of study quality assessment (score 0–9) yielded a score of 7.0 or above for 15 studies (Table S2).

**Table S1:** Descriptive characteristics of included cohort studies.

Author	Publication year	Country	Participants	Measurements for physical activity	Number of diabetes	Age at baseline (years)	Duration (years)	Risk of outcomes (95% CI)	
Batty, et al^1^	2002	UK	The study was conducted in 6408 male British Civil Servants who underwent an oral glucose tolerance test at study entry.	The active group included men who engaged in vigorous sports such as swimming, cycling and athletics; the moderately active group comprised men who participated in active hobbies such as gardening, home maintenance and woodwork; the inactive group included those men who reported no such physical exertion.	6408	520.51	25	All-cause mortality: Active: 1.0 (reference) Moderate: 1.59 (1.1–2.4) Inactive: 1.65 (1.1–2.5) CHD mortality: Active: 1.0 (reference) Moderate: 2.47 (1.1–5.4) Inactive: 3.6 (1.6–8.0) Other CVD mortality Active: 1.0 (reference) Moderate: 1.35 (0.5–3.5) Inactive: 2.56 (0.6–4.2)	
Tanasescu, et al^2^	2003	USA	The HPFS was conducted in 51 529 male health professionals aged 40 to 75 years in 1986 and living in all 50 US states.	The time spent at each activity in hours per week was multiplied by its typical energy expenditure, expressed in METs, then summed over all activities to yield a MET-hour score. Participants were classified base on quintile of MET-hour score.	2803	40–75	14	CVD events: Q1: 1.0 (reference) Q2: 0.91 (0.63, 1.31) Q3: 0.68 (0.45, 1.02) Qu 4: 0.76 (0.51, 1.14) Q5: 0.72 (0.47, 1.09) CVD mortality: Q1: 1.0 (reference) Q2: 0.71 (0.40, 1.28) Q3: 0.29 (0.14, 0.63) Q4: 0.53 (0.27, 1.02) Q 5: 0.62 (0.32, 1.23)	
Hu, et al 3	2005	Finnish	The study was conducted in 3,708 Finnish patients with type 2 diabetes aged 25–74 years.	Physical activities were merged three categories: 1) low was defined as subjects who reported light levels of occupational, commuting (<1 min), and leisure-time physical activity; 2) moderate was defined as subjects who reported only one of the all three types of moderate to high physical activity; and 3) high was defined as subjects who reported two or three types of moderate to high physical activity	3,708	25–74	18.7	All-cause mortality: Low physical activity: 1.0 (reference) Moderate: 0.61 (0.51–0.73) High: 0.55 (0.47–0.66) CVD mortality: Low physical activity: 1.0 (reference) Moderate: 0.57 (0.46–0.72) High: 0.54 (0.43–0.67)	
Jonker, et al 4	2006	USA	The Framingham Heart Study cohort consisted of 5,209 respondents (46% male) aged 28.62 years residing in Framingham, Massachusetts, between 1948 and 1951.	Time spent at each activity in hours per week was multiplied by its metabolic cost (based on the oxygen consumption required for that activity). These weighted hours were added up to get a total daily physical activity score. Participants were classified based on tertiles of the daily physical activity scores: low <30), moderate (30–33), and high >33) physical activity level.					5209
Trichopoulou, et al^5^	2006	Greece	The Greek arm of the European Prospective Investigation into Cancer (EPIC) and Nutrition was conducted in 28572 adult volunteers from 1993 to mid-2004, in the Greek arm of the European Prospective Investigation into Cancer and Nutrition.	A metabolic equivalent index was computed by assigning a multiple of resting metabolic rate to each activity metabolic equivalent task (MET value) and all MET-hour products were summed to estimate daily physical activity. Participants were classified based on quintile of MET-hour score.					1013
Tielemans, et al 6	2013	European	The EURODIAB Prospective Complications Study is a cohort including 3,250 male and female patients with type 1 diabetes.	Physically inactive participants were considered to be those who reported walking <1.5 kilometers on an average weekday, no regular bicycling and no participation in sports. Participants who reported walking ≥1.5 kilometers on an average weekday, regular bicycling or played any sport were considered to be the physically active group.					2185
Li, et al^7^	2013	Taiwan, China	A national sample of adults (18 years or older) with self-reported physician-diagnosed diabetes, who participated in the 2001 National Health Interview Survey in Taiwan (N = 797).	The PCS was aggregated from z-score transformations of the eight dimensions and then standardized to a mean of 50 and a standard deviation of 10. Then participants were classified based on quartiles of PCS.					797
Blomste,et al^8^	2013	Australasia, Asia, Europe and North America	The study was conducted in 11 140 patients in the ADVANCE (Action in Diabetes and Vascular Disease: Preterax and Diamicron modified release Controlled Evaluation) trial.	For primary analyses, participants were divided into who were sedentary or undertook only mild physical activity in the prior week and those who participated in at least one session of moderate or vigorous physical activity >15 min. In subsequent analyses authors sought to clarify whether participation in mild physical activity was beneficial. Then they compared the outcomes of patients who undertook only mild exertion with those who were entirely sedentary and those who undertook moderate or vigorous exercise at the week prior to randomization.	11 140	65.8 ±6.4	5	All-cause mortality Sedentary: 1.0 (reference) Mild: 0.86 (0.72–1.02) Moderate and vigorous: 0.74 (0.62- 0.88) Cardiovascular mortality Sedentary: 1.0 (reference) Mild: 1.06 (0.90–1.26) Moderate and vigorous: 0.82 (0.68- 0.97)	
Moe, et al 9	2013	Norway	The HUNT Study is a large population-based health survey in Nord- Trondelag County conducted in inhabitants aged 20 years or older	Participants performing 0.1–1.9 hours of leisure time physical exercise per week were defined as physically inactive; participants performing 1.0–1.9 hours of leisure time physical exercise per week were defined as mild active; and participants performing . 2.0 hours of leisure time physical exercise per week were defined as moderate active.	503	63.7±15.4	23.8	Cardiovascular mortality: Without diabetes* inactive:1.0 (reference) Diabetes without medication* inactive: 1.65 (1.34–2.03) Diabetes without medication*mild: 1.44 (1.13–1.83) Diabetes without medication*moderate: 0.99 (0.68–1.45) Diabetes with medication*inactive: 2.46 (2.08–2.92) Diabetes with medication*mild: 2.64 (2.14- 3.25) Diabetes with medication*moderate: 1.58 (1.21–2.05)	
Sone, et al^10^	2013	Japan	The present analysis was conducted as part of the Japan Diabetes Complications Study (JDCS), a multicenter prospective study of the incidence of and risk factors for complications among 2,033 Japanese patients with type 2 diabetes aged 40–70 years with HbA1c levels ≥6.5%.	Participants were divided into 3 groups based on tertile of LTPA, which was assessed at baseline by a self-administered questionnaire.	1702	58.5±6.9	8.05	All-cause mortality T1: 1.0 (reference) T2: 0.88 (0.47, 1.64) T3: 0.47 (0.22, 0.99) CHD or stroke T1: 1.0 (reference) T2: 0.96 (0.61, 1.50) T3: 0.68 (0.42, 1.11)	
Williams, et al^11^	2014	USA	The National Walkers' and Runners' Health Studies was conducted to assess the relationships of running and walking to mortality in diabetic subjects.	Participants were divided into 3 groups according to MET: 1) falling short of the current exercise recommendations for health (<450 MET·min·wk-1 = 1.07 MET·h·d-1 ), 2) achieving the exercise recommendations (450–750 MET·min·wk-1 = 1.07–1.8 MET·h·d-1 ), and 3) exceeding the recommendations.	2160	57.17±13.99	9.84	Total mortality T1: 1.0 (reference) T2: 0.925 (0.676, 1.247) T3: 0.635 (0.489, 0.821) CVD mortality T1: 1.0 (reference) T2: 0.76 (0.51, 1.12) T3: 0.54 (0.39, 0.75)	
Zethelius, et al^12^	2014	Sweden	The study was conducted in 15,462 female and male patients with type- 2 diabetes registered in the NDR, with data available for all analyzed variables.	Five levels of PA were classified: never (level 1), less than 1 time per week (level 2), 1–2 times per week (level 3), regular 3–5 times per week (level 4), or daily (level 5).	15,462	30–72	5	All-cause mortality: Low vs. high: 2.91 (2.08–4.07) CVD: Low vs. high: 2.54 (1.98–3.27)	
Martinez- Gomez, et al^13^	2015	Spain	A cohort of 4,008 persons representative of the non-institutionalized population aged 60 years and older in Spain.	Physical activity was assessed with a global questionnaire that asked participants to rate their level of physical activity in 4 categories as inactive, less active, moderately active and very active in comparison with their age-peers.	611	72.1± 7.6	8.3	Inactive: 1.0 (reference) Less active: 0.72 (0.49, 1.08) Moderately active: 0.47 (0.31, 0.70) Very active: 0.52 (0.32, 0.82)	
Lear, et al^14^	2017	17 countries	Participants were recruited participants from 17 countries aged between 35 and 70 years who intended to live at their current address for at least another 4 years.	Total physical activity was categorized as low (<600 MET × minutes per week), moderate (600–3000 MET × minutes per week), and high (>3000 MET × minutes per week) physical activity, corresponding to less than 150 minutes per week, 150–750 minutes per week, and more than 750 minutes per week of moderate intensity physical activity.	12740	35–70	6.9	High PA vs low PA: 0.68 (0.57–0.82) Moderate PA vs low PA: 0.80 (0.67–0.95)	
Shin, et al 15	2018	UK	The NHIS-HEALS cohort comprised a nationally representative random sample of 514,795 individuals, which accounted for 10% of the entire population who were aged between 40–79 years in 2002 and 2003	Frequency of exercise was determined at study entry with a questionnaire. Participants were requested to estimate the exercise frequency per week at baseline, and were classified as exercising on 0, 1–2, 3–4, 5–6, and 7 days per week.	6923	12	Not reported	Exercising 0 days per week:1.0 (reference) Exercising 1–2 days per week:0.79 (0.74– 0.85) Exercising 3–4 days per week:0.74 (0.67– 0.81) Exercising 5–6 days per week:0.67 (0.56– 0.79) Exercising 7 days per week:0.87 (0.80–0.94)	
Zhao^16^	2020	USA	The study included data from the National Health Interview Survey (NHIS) conducted since 1957 by the US Centers for Disease Control and Prevention and the National Center for Health Statistics through the US Census Bureau. A cohort of 479,856 adults aged 18 years or older in USA.	Participants were classified into one of four groups depending on whether they met each of the recommended guidelines: insufficient activity (insufficient aerobic and muscle strengthening activities), aerobic physical activity only (recommended aerobic activity and insufficient muscle strengthening activity), muscle strengthening only (insufficient aerobic activity and recommended muscle strengthening activity), and both (recommended aerobic and muscle strengthening activities).	40440	Not reported	8.75	Insufficient aerobic or muscle strengthening:1.00 (reference) Muscle strengthening only:0.98 (0.87 to 1.10) Aerobic only:0.68 (0.64 to 0.72) Aerobic and muscle Strengthening:0.60 (0.53 to 0.67)	

Abbreviations: CI, confidence interval; NOS, Newcastle-Ottawa quality assessment; Mets, metabolic equivalents; PA, physical activity; PCS, physical component summary; T1DM, type 1 diabetes mellitus; T2DM, type 2 diabetes mellitus.

**Table S2:** Quality assessment of individual studies using Newcastle-Ottawa Scale

Reference	Selection				Comparability	Outcome			Overall Quality		
	Representative of cases	Selection of Controls	Exposure ascertainment (weight change)	No history of Disease	Comparable on confounders	Outcome assessment (by medical record or doctors)	Adequate follow-up time (≥ 5 years)	Follow-up rate (> 80%)	Overall Quality
Batty, 2002	1.0	1.0	0.5	1.0	1.5	1.0	1.0	1.0	8.0
Tanasescu, 2003	0.5	1.0	0.0	1.0	2.0	1.0	1.0	1.0	7.5
Hu, 2005	1.0	1.0	1.0	1.0	2.0	1.0	1.0	1.0	9.0
Jonker, 2005	0.5	1.0	1.0	1.0	1.5	1.0	1.0	1.0	8.0
Trichopoul, 2006	1.0	1.0	1.0	1.0	2.0	1.0	1.0	1.0	9.0
Tielemans, 2013	0.5	1.0	1.0	1.0	2.0	1.0	0.5	0.5	7.5
Li, 2013	0.5	1.0	1.0	1.0	1.5	1.0	1.0	1.0	8.0
Blomster, 2013	1.0	1.0	1.0	0.5	2.0	1.0	1.0	1.0	8.5
Moe, 2013	1.0	1.0	1.0	1.0	2.0	1.0	1.0	1.0	9.0
Sone, 2013	1.0	1.0	1.0	1.0	1.5	1.0	1.0	0.5	8.0
Zethelius, 2014	1.0	1.0	1.0	0.5	1.0	0.5	0.5	1.0	6.5
Williams, 2014	0.5	1.0	0.5	1.0	2.0	1.0	1.0	1.0	8.0
Martinez, 2015	1.0	1.0	0.5	0.5	2.0	1.0	1.0	0.5	7.5
Lear, 2017	1.0	1.0	1.0	0.5	1.5	0.5	1.0	1.0	7.5
Shin, 2018	0.5	1.0	1.0	0.5	1.0	0.5	1.0	1.0	6.5
Zhao, 2020	1.0	1.0	1.0	0.5	1.5	1.0	1.0	1.0	8.0

### Overall analyses

**Figure 1:**
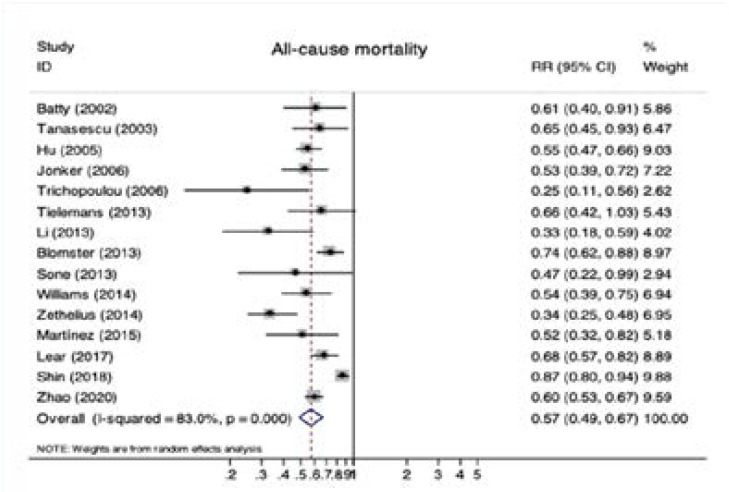
Forest plot of physical activity and risk of all-cause mortality. Abbreviations: RR, risk ratios; CI, confidence interval.

**Figure 2:**
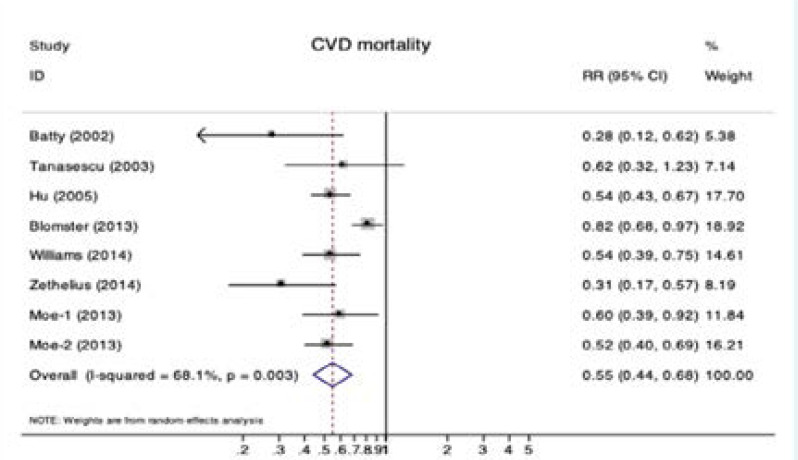
Forest plot of physical activity and risk of CVD mortality. Abbreviations: RR, risk ratios; CI, confidence interval; Moe-1 means outcomes for diabetes patients without medication, and Moe-2 means outcomes for diabetic patients with medication.

**Figure S2:**
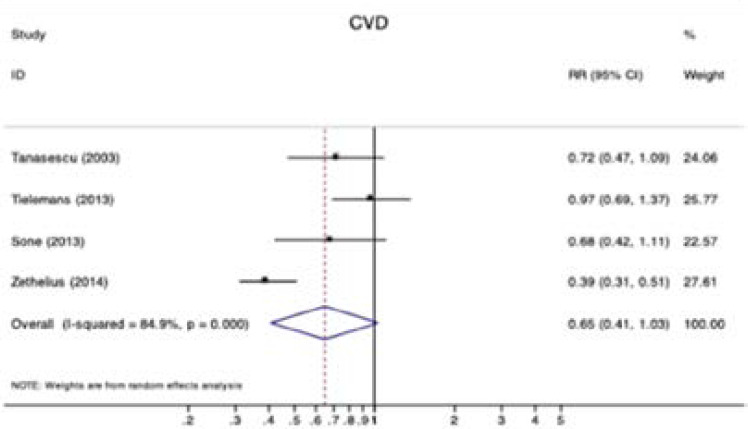
Forest plot of physical activity and risk of CVD.

Fifteen observational studies were included in the analysis of diabetes and all-cause mortality. Significant reductions in all-cause mortality were observed for diabetic patients with physical activity (RR 0.57, 95% CI 0.49–0.67; P < 0.001; Figure 1). Obvious heterogeneity was detected among these studies (I2 = 83.0%; P < 0.001 for heterogeneity; Figure 1). Furthermore, pooled estimates of seven studies showed that diabetic patients with physical activity had lower risk for CVD mortality (RR 0.55, 95% CI 0.44–0.68; P < 0.001; Figure 2) with moderate heterogeneity (I2 = 68.1%; P = 0.003 for heterogeneity; Figure 2). There were four studies reported CVD evens in diabetic patients with active or inactive physical activity and the pooled RR for CVD was 0.58 (95% CI 0.49–0.69; Figure S2) by a random effects model (I2 = 84.9%; Figure S2).

### Sensitivity and subgroup analyses

**Figure S3:**
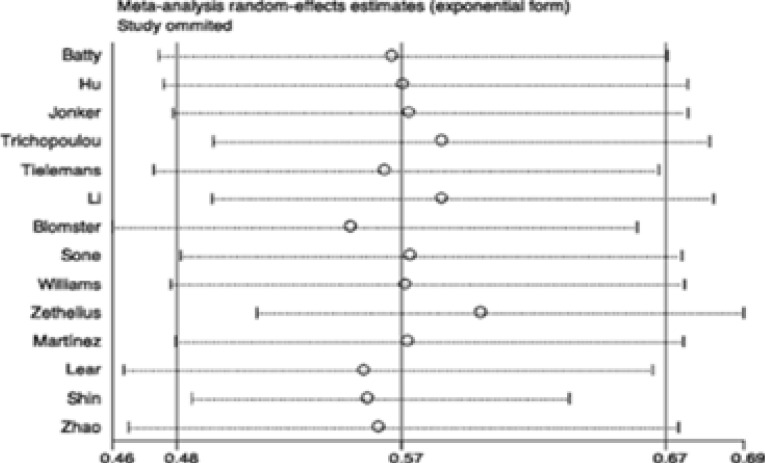
Sensitivity analysis of studies for all-cause mortality.

**Figure S4:**
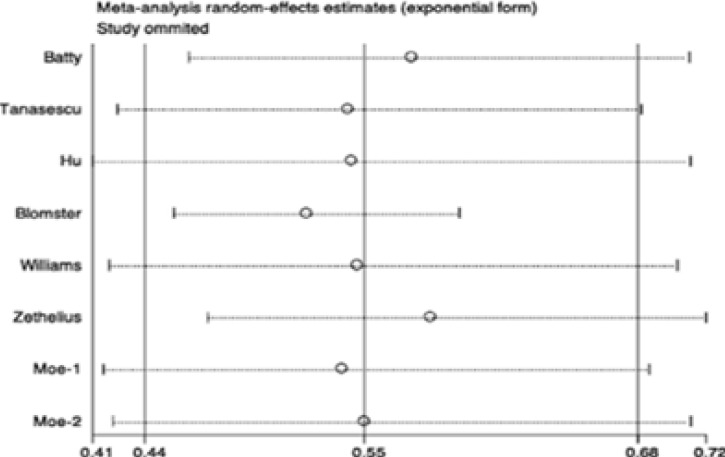
Sensitivity analysis of studies for CVD mortality.

**Figure S5:**
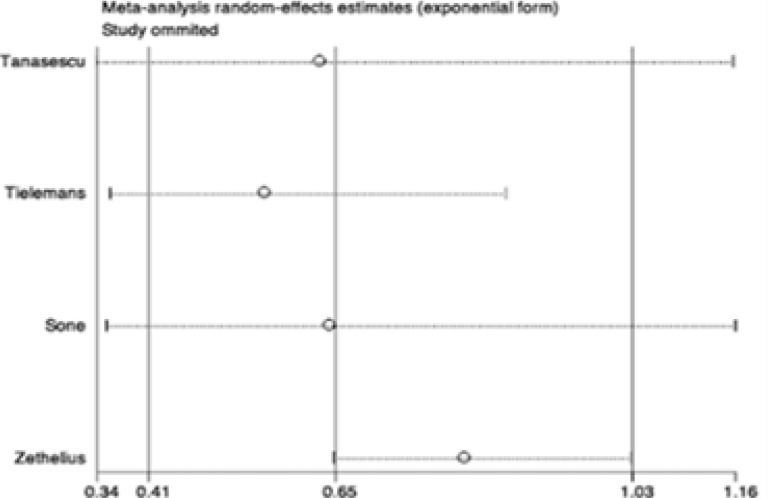
Sensitivity analysis of studies for CVD.

Sensitivity and subgroup analyses were conducted to examine the stability of the primary results. In the sensitivity analyses, through omission of any individual study, the results for all-cause and CVD mortality were not significantly altered (Figure S3–S4). However, the results for CVD were changed in the sensitivity analysis (Figure S5), which may due to limited studies were included. We further conducted subgroup analyses for all-cause mortality stratified by age, study location, number of participants and case, follow-up of cohort studies, HbA1c, type and duration of diabetes. Results of any subgroup were consistent with our overall findings (Table S3). Importantly, the reductions in all-cause mortality were more significant in diabetic patients with old age (> 60 years) (RR 0.46, 95% CI 0.29–0.75; P = 0.002; Table S3), higher BMI (BMI ≥ 28) (RR 0.53, 95% CI 0.42–0.69; P < 0.001; Table S3) and shorter duration of diabetes (RR 0.45, 95% CI 0.24–0.84; P < 0.001; Table S3). There were eight studies conducted in T2DM and one study conducted in type 1 diabetes mellitus (T1DM). The remains didn't provide the information of types of diabetes. The summary RR for T2DM was 0.56 (95% CI 0.46–0.68; P < 0.001; Table S3) and the RR for T1DM was 0.66 (95% CI 0.42–1.03; P = 0.007; Table S3). Furthermore, there were four studies provided data of HbA1c and the average of HbA1c in these studies were more than 7%. The pooled RR of these 4 studies was 0.54 (95% CI 0.35–0.84; P < 0.001; data was not shown in Table). We didn't perform subgroup analysis for CVD mortality and events because the limited studies were included in each subgroup, which may provide some misleading information.

**Table S3:** Subgroup analyses of relative risk of all-cause mortality

	n	RR (95% CI)	P^1^	I^2^ (%)	P^2^
All studies	15	0.57 (0.49–0.67)	< 0.001	83.0	< 0.001
Age					
> 60 years	4	0.46 (0.29–0.75)	0.002	77.4	0.004
≤ 60 years	9	0.58 (0.47–0.72)	< 0.001	86.7	< 0.001
Study location					
Asia	2	0.38 (0.24–0.60)	< 0.001	0.0	0.47
North America	2	0.58 (0.46–0.73)	< 0.001	0.0	0.40
Europe	7	0.54 (0.39–0.74)	< 0.001	89.8	< 0.001
Africa	2	0.59 (0.53–0.66)	0.002	0.0	0.55
Mixed	2	0.71 (0.63–0.81)	< 0.001	0.0	0.51
Number of participants					
> 3000	7	0.61 (0.50–0.74)	< 0.001	90.7	< 0.001
≤3000	8	0.54 (0.45–0.64)	< 0.001	16.1	0.303
Number of death					
> 300	7	0.61 (0.50–0.74)	< 0.001	90.7	< 0.001
≤300	8	0.54 (0.45–0.64)	< 0.001	16.0	0.304
Follow-up of cohort studies (years)					
≥ 9	7	0.64 (0.52–0.78)	< 0.001	83.9	< 0.001
< 9	8	0.51 (0.40–0.64)	< 0.001	73.4	< 0.001
BMI					
≥ 28	5	0.53 (0.42–0.69)	< 0.001	78.4	0.001
< 28	9	0.59 (0.48–0.74)	< 0.001	77.5	< 0.001
Type of diabetes					
T2DM	7	0.56 (0.46–0.68)	< 0.001	68.7	0.004
T1DM	1	0.66 (0.42–1.03)	0.07	-	-
Duration of diabetes (years)					
≥ 8	4	0.50 (0.35–0.71)	< 0.001	29.6	0.234
< 8	3	0.45 (0.24–0.84)	0.012	90.5	< 0.001

**Abbreviations**: CI, confidence interval; CC, cortical cataract posterior; PSC, posterior sub capsular; NS, nuclear sclerosis.

N, the number of studies (the number of studies is not always equal to the total because of missing information in some publications or subgroups in original studies).

1 P for text

2 P for heterogeneity between subgroups with analysis

### Publication bias

As shown in Table S4, no publication bias was observed according to Egger's and Begg's test in studies of analysis for mortality from all causes and CVD, and CVD events (all P > 0.05).

**Table S4:** Assessment for heterogeneity and publication bias

		Tests for Heterogeneity				Tests for Publication Bias	
	N	RR (95% CI) by random-effect model	RR (95% CI) by fixed-effect model	P value for heterogeneity	I^2^ (%)	P value of the Egger test	P value of the Begg test
All-cause mortality	14	0.57 (0.49–0.67)	0.69 (0.66–0.73)	<0.001	83.0	0.13	0.51
CVD mortality	8	0.55 (0.44–0.68)	0.61 (0.55–0.68)	0.003	68.1	0.06	0.54
CVD	4	0.65 (0.41–1.03)	0.58 (0.49–0.69)	<0.001	84.9	0.31	1.00

Abbreviations: RR, relative risk; CI, confidence interval.

## Discussion

In this meta-analysis involving 155,203 diabetic patients, we concluded that physical activity reduced risk for total and CVD mortality among patients with diabetes. Furthermore, the reductions were more significant in diabetic patients with old age (> 60 years), obesity and shorter duration of diabetes.

Several high-quality meta-analyses summarized the studies conducted in general population and conducted that physical activity may reduce the risk of all-cause, cardiovascular and cancer mortality, and incident type 2 diabetes^35^–^41^. We firstly summarized the studies conducted in diabetic patients and conducted that physical activity could reduce the risk of total and CVD mortality, as well as CVD events among diabetes. It may contributed by the beneficial effect of physical activity on several indices of cardio metabolic diseases including body weight^42^, body fat distribution 42–44, blood pressure 45, blood lipids^43^,^46^, insulin resistance47,48, endothelial function 49 and cardio-respiratory fitness^42^,^44^. It was also reported that moderate or high levels of physical activity were associated with a significantly reduced risk of total and CVD mortality among adults with diabetes, independent of age, education, BMI, blood pressure, total cholesterol, and smoking^12^. Aerobic and resistance training improve insulin action and plasma glucose (PG), lipids, blood pressure and cardiovascular risk 50. Regular exercise is necessary for continuing benefit. Therefore, diet control and lifestyle interventions are recommended as the first-line treatments for diabetes 11. Schellenberg et al also found that lifestyle interventions effectively decrease the incidence of type 2 diabetes in high-risk patients 51. However, in patients who already have type 2 diabetes, there is no evidence of reduced all-cause mortality and insufficient evidence to suggest benefit on cardiovascular and micro vascular outcomes 51. We, therefore, tried to further analyze the association in diabetic patients stratified by age, BMI, HbA1c and duration of diabetes. The results showed that the reductions of total mortality were more significant in diabetic patients with old age (> 60 years), obesity and shorter duration of diabetes. It was reported that older adults> 65 years old) are the least physically active age group 52. In advanced age, physical activity is effective at mitigating sarcopenia, restoring robustness, and preventing/delaying the development of disability 53. As we discussed previously, BMI is an independent risk factor of diabetes and CVD 9, therefore physical activity may have more protective effects on diabetic patients with obesity.

The significant reduction of total mortality in the patients with shorter duration of diabetes could, in part, be due to the protection of physical activity on β cell from further failure induced by lipotoxicity in early stage. Besides, the physical activity did not reduce the total mortality in patients with T1DM 17, which may due to the hypoglycemia during the immediate post exercise period 54.

There are several limitations for the current study. Firstly, our meta-analysis was performed on summary data, thus leading to a relatively poor accuracy of assessment compared with individual-level analyses. Secondly, our study was limited to studies reported in English. Furthermore, significant heterogeneity was detected among all studies, may due to the different measurements of physical activity. Finally, we cannot identify the best way and the most appropriate amount physical activity for diabetes to achieve the greatest benefit.

## Conclusion

The present study added to the literature by confirming the association between physical activity and risk for total and CVD mortality among diabetic patients, in particular in diabetic patients with old age (> 60 years), obesity and shorter duration of diabetes. It may provide some information for policymakers and future guidelines. Future study is needed to summarize the dose-response association of different kinds of physical activity and health outcomes in patients with diabetes.

## Data Availability

The datasets used and/or analyzed during the current study are available from the corresponding author on reasonable request.

## Abbreviations

BMI: Body mass index
CIs: confidence intervals
CVD: cardiovascular diseases
HbA1c: glycated haemoglobin A1c
HR: hazard ratios
IGT: impaired glucose tolerance
NOS: Newcastle-Ottawa scale
OR: odds ratios
PG: plasma glucose
RRs: relative risks
T1DM: type 1 diabetes mellitus
T2DM: type 2 diabetes mellitus

## Findings

This study received no specific funding for this work.
